# Multisystem Inflammatory Syndrome in Children and Long COVID: The SARS-CoV-2 Viral Superantigen Hypothesis

**DOI:** 10.3389/fimmu.2022.941009

**Published:** 2022-07-07

**Authors:** Magali Noval Rivas, Rebecca A. Porritt, Mary Hongying Cheng, Ivet Bahar, Moshe Arditi

**Affiliations:** ^1^ Department of Pediatrics, Division of Infectious Diseases and Immunology, Guerin Children’s at Cedars-Sinai Medical Center, Los Angeles, CA, United States; ^2^ Infectious and Immunologic Diseases Research Center (IIDRC) and Department of Biomedical Sciences, Cedars-Sinai Medical Center, Los Angeles, CA, United States; ^3^ Department of Computational and Systems Biology, School of Medicine, University of Pittsburgh, Pittsburgh, PA, United States; ^4^ Smidt Heart Institute, Cedars-Sinai Medical Center, Los Angeles, CA, United States

**Keywords:** SARS-CoV-2, superantigen, MIS-C multisystem inflammatory syndrome in children, long COVID, superantigen-like motif, neurotoxin-like segment, post-acute sequelae of COVID-19 (PASC)

## Abstract

Multisystem inflammatory syndrome in children (MIS-C) is a febrile pediatric inflammatory disease that may develop weeks after initial SARS-CoV-2 infection or exposure. MIS-C involves systemic hyperinflammation and multiorgan involvement, including severe cardiovascular, gastrointestinal (GI) and neurological symptoms. Some clinical attributes of MIS-C—such as persistent fever, rashes, conjunctivitis and oral mucosa changes (red fissured lips and strawberry tongue)—overlap with features of Kawasaki disease (KD). In addition, MIS-C shares striking clinical similarities with toxic shock syndrome (TSS), which is triggered by bacterial superantigens (SAgs). The remarkable similarities between MIS-C and TSS prompted a search for SAg-like structures in the SARS-CoV-2 virus and the discovery of a unique SAg-like motif highly similar to a Staphylococcal enterotoxin B (SEB) fragment in the SARS-CoV-2 spike 1 (S1) glycoprotein. Computational studies suggest that the SAg-like motif has a high affinity for binding T-cell receptors (TCRs) and MHC Class II proteins. Immunosequencing of peripheral blood samples from MIS-C patients revealed a profound expansion of TCR β variable gene 11-2 (TRBV11-2), which correlates with MIS-C severity and serum cytokine levels, consistent with a SAg-triggered immune response. Computational sequence analysis of SARS-CoV-2 spike further identified conserved neurotoxin-like motifs which may alter neuronal cell function and contribute to neurological symptoms in COVID-19 and MIS-C patients. Additionally, autoantibodies are detected during MIS-C, which may indicate development of post-SARS-CoV-2 autoreactive and autoimmune responses. Finally, prolonged persistence of SARS-CoV-2 RNA in the gut, increased gut permeability and elevated levels of circulating S1 have been observed in children with MIS-C. Accordingly, we hypothesize that continuous and prolonged exposure to the viral SAg-like and neurotoxin-like motifs in SARS-CoV-2 spike may promote autoimmunity leading to the development of post-acute COVID-19 syndromes, including MIS-C and long COVID, as well as the neurological complications resulting from SARS-CoV-2 infection.

## Introduction

COVID-19, an infectious disease caused by the severe acute respiratory syndrome coronavirus 2 (SARS-CoV-2), became a major pandemic in 2020. The spectrum of COVID-19 clinical manifestations is broad, and SARS-CoV-2 infected individuals can present as asymptomatic or with mild symptoms such as fever, fatigue, sore throat, runny nose, and coughing. Severe COVID-19 develops in some individuals and is characterized by interstitial pneumonia, hypoxemia, and acute respiratory distress syndrome (ARDS), which may be lethal (1, 2). Advanced age, male sex and underlying conditions such as diabetes, obesity, active cancer, chronic lung and kidney diseases and cardiovascular diseases are risk factors associated with severe disease development (3–5). Children are less severely affected by COVID-19 than adults and severe respiratory deteriorations are rare in SARS-CoV-2 infected children (6). This variation in clinical presentation might be explained by more rapid and potent innate and adaptive immune responses, the lower rates of comorbidities associated with severe COVID-19 in children and, to a lesser extent, the presence of cross-reactive T cells triggered by previous coronavirus infections (7, 8). However, weeks after SARS-CoV-2 infection, a small fraction of children develop a post-infectious febrile pediatric hyperinflammatory entity called multisystem inflammatory syndrome in children (MIS-C) (9–14). MIS-C affects multiple organs, including the cardiovascular system, the gastrointestinal (GI) tract, and the kidneys, and may lead to the development of neurological symptoms (9–14). Because of some shared clinical manifestations, MIS-C has been initially described as a Kawasaki disease (KD)-like syndrome. However, MIS-C demographic features, clinical, laboratory and immunopathological findings differ from those of KD (15–17). A similar MIS developing in adults of 21 years and older, called MIS-A, has also been reported by the CDC (18). Two years after the initial reports of MIS-C, it remains unclear why some children develop MIS-C after SARS-CoV-2 infection, whereas others successfully clear the infection without the complications of long-lasting sequelae.

## MIS-C and SARS-CoV-2-Induced Hyperinflammation and Toxic Shock Syndrome

MIS-C can lead to severe health complications, such as cardiogenic shock and multiorgan failure, and frequently requires admission to the intensive care unit (ICU). It was initially reported in a small cohort of English children as a hyperinflammatory shock presenting with clinical features similar to KD, KD shock, cytokine storm, and toxic shock syndrome (TSS) (10). Following this initial report, MIS-C cases were described in multiple cities that were strongly affected by COVID-19 (9, 11, 13, 14, 19, 20). MIS-C is now understood as a post-infectious hyperinflammatory response with an autoimmune component that develops in children who either had a SARS-CoV-2 positive test or contact with a SARS-CoV-2 infected individual in the weeks preceding diagnosis (21). Increased incidence of MIS-C cases is usually observed 2 to 5 weeks after the peak of SARS-CoV-2 infections in affected geographic areas (22).

Persistent fever, conjunctivitis, skin rash, myocardial dysfunction, hypotension or shock and temporary development of coronary artery dilatations are common clinical complications associated with MIS-C (9–12, 14). These features overlap with symptoms of KD, a febrile inflammatory and systemic vasculitis of unknown etiology that leads to coronary artery aneurysms in young children (23–25). However, distinct etiology, epidemiological and demographic data, clinical symptoms and laboratory results indicated that MIS-C likely represents a different entity than KD (15–17, 26). Compared with KD, which is mostly reported in younger children (< 5 years) and has highest incidence in Pacific Islander and Asian populations (23–25), MIS-C incidence is higher in children from non-Hispanic Black and Hispanic or Latino ethnicities, with a median age of 9 years (21). Some MIS-C patients develop mild coronary artery aneurysms, which, unlike the aneurysms that occur in KD patients, are transient and regress over time (27, 28). Furthermore, clinical and laboratory findings indicate more pronounced lymphopenia and thrombocytopenia and higher neutrophil counts and C-reactive protein (CRP) levels in MIS-C individuals than in KD patients (9, 11, 12, 20).

Children with MIS-C also exhibit severe abdominal pain and GI symptoms, myocardial cardiogenic shock, neurological findings, and kidney involvement, which are very uncommon in KD but frequently associated with TSS (13, 14, 16, 29–31). TSS is triggered by bacterial superantigens (SAgs). SAgs bind MHC class II (MHC II) molecules on antigen presenting cells (APCs) and T cell receptors (TCRs) bearing specific Vβ fragments, which leads to the activation of up to 30% of T cells and uncontrolled release of proinflammatory cytokines (cytokine storm) (32, 33). Initially linked to the use of tampons by menstruating young women, TSS is also reported after postsurgical infections, and may affect healthy children and male individuals (34, 35). The clinical manifestations of TSS also include acute fever, hypotension, and multisystem involvement, including the GI tract and cardiac tissues, and skin rash which progresses to skin desquamation 1 to 2 weeks after disease onset (35). TSS is also associated with neurological symptoms, such as headache and confusion, which are also reported during MIS-C (35, 36).

SAg activity has also been observed in several viruses, including Epstein-Barr virus (EBV) (37), Herpes Simplex Virus type I (HSV1) (38), Ebola (39), human endogenous retroviruses (40), cytomegalovirus (41), HIV-1 (42) and rabies virus (43). The viral etiology of MIS-C and its overlapping clinical features with TSS prompted us to search for a SAg-like structure in SARS-CoV-2 (44).

## Discovery of a Superantigen-Like Motif in SARS-CoV-2 Spike

**Figure 1:**
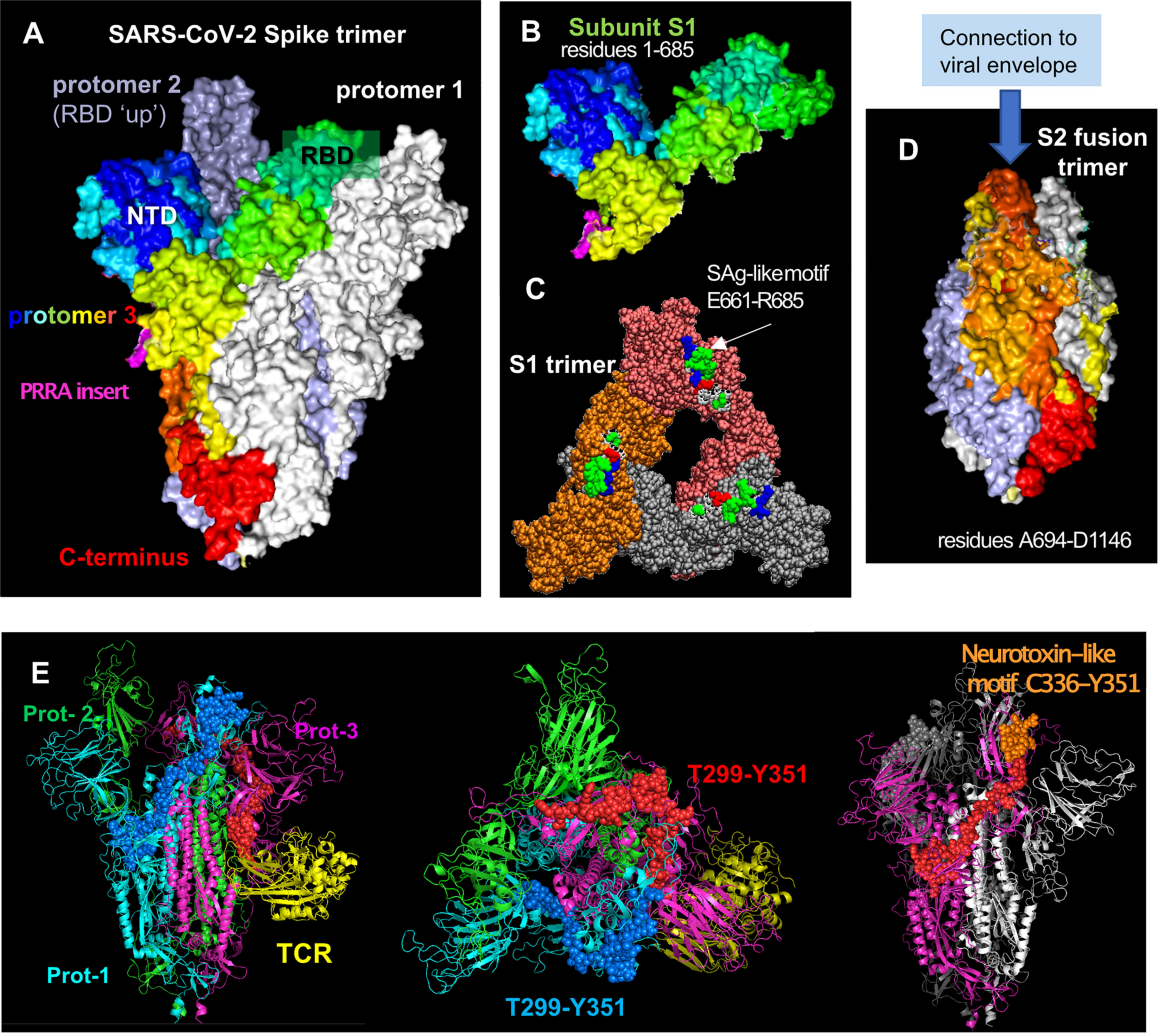
SARS-CoV-2 spike glycoprotein structure, its structural subunits, putative SAg and neurotoxin-like motifs. **(A)** The SARS-CoV-2 spike trimer in the pre-fusion state, where one of the protomers is shown in spectral colors from *blue* (N-terminal domain, NTD) to *red* (C-terminus), and the other two protomers are shown in white and gray. Each protomer has a Receptor-Binding Domain (RBD) that can assume up and down conformations in the receptor-bound and unbound states. **(B)** Structure of S1 subunit, shown for the spectrally colored protomer, in the same format and perspective as in **(A)**. *Pink* color showed the “PRRA” insert unique to SARS-CoV-2. **(C)** S1 trimer after shedding of the S2 trimer, shown from top. Each protomer is shown in a different color (*orange, brick*, and *gray*). The SAg-like motifs (E661 to R685) in the SARS-CoV-2 spike S1 trimer are shown in van der Waals (VDW) format; *white, green, red, and blue* represent hydrophobic, hydrophilic, acidic and basic residues. **(D)** S2 subunits after cleavage, forming a fusion trimer [same color and format as in **(A)**]. **(E)** Neurotoxin motifs on the spike glycoprotein. Side (*left panel*) and top (*middle panel*) views of the spike in the presence of a bound TCR (*yellow ribbon*) are shown. The spike protomers are colored *green, cyan and magenta* in this case, and the neurotoxin motif [residues 299-351; reported in (44)] belonging to the *cyan* and *magenta* protomers are displayed in *blue* and *red* spheres, respectively. Note that the portion C336-Y351 (*orange* spheres on the *right panel*) is exposed to interact with the host cell receptor or substrates. The homology model [44] constructed based on the cryo-EM structure resolved by Wrapp et al. (2020) (PDB id: 6vsb) has been used in the ribbon diagrams.

Spike glycoproteins are expressed as trimers at the surface of SARS-CoV-2, each monomer being composed of two subunits, S1 and S2 (**Figures 1A–D**), which have different functions (45). S1 binds to the human angiotensin converting enzyme 2 (ACE2) at the surface of the target cell, and S2 mediates the fusion of the viral and host cell membranes (45, 46). After SARS-CoV-2 spike protein binds to ACE2, the spike is sequentially cleaved, first at the S1/S2 junction and then at the so-called S2’ cleavage on the S2 subunit, by human proteases (furin and transmembrane protease serine 2 (TMPRSS2) at the respective sites) which prompt the spike for membrane fusion and viral entry to the host cell (45, 46).

The SARS-CoV-2 spike subunit S1 has an insertion of four amino acids, P_681_RRA_684_ (PRRA), adjacent to the cleavage site R_685_↑S_686_ at the interface between the S1 and S2 subunits (**Figures 1A, B** *; magenta*) (45). The polybasic segment PRRA is unique to SARS-CoV-2 and the SARS-like subfamily of β-coronaviruses (45). Using computational analysis, we found that the PRRA insert is part of a motif of 25 amino acids, E661-R685, that has sequence and structure features similar to a segment of the SAg Staphylococcal enterotoxin B (SEB) (44). In particular, the fragment T_678_NSPRRARSV_687_ in the spike exhibits 30% sequence identity to the SEB superantigenic motif T_150_N–KKKATV_157_, in addition to sharing a polybasic stretch (spike RRAR_685_ aligned against SEB KKKA_155_). Furthermore, the inverted SEB sequence **Q_158_VTAKKKNT_150_** can be structurally aligned against the spike sequence **Q_677_TNSPRRAR_685_,** as shown in earlier work (44). Note that R681 in this segment is susceptible to mutations, e.g. P681H and P681R found in different SARS-CoV-2 variants. Our simulations further indicated that this SAg-like motif may bind to TCRs and MHC Class II (44). It is important to note that this SAg-like motif is immediately adjacent (sequentially) to the furin-like cleavage site R_685_↑S_686_ (45). The polybasic nature of the furin cleavage site itself is critically important for recognition of that site by the acidic epitope of furin, and in fact, viral mutations that delete the furin cleavage site, which happens to include the SAg-like motif, have been shown to reduce SARS-CoV-2-induced pathogenesis in mice and hamsters (47, 48). These observations may perhaps suggest a potential involvement of the SAg-like motif as well as the furin-binding site in the pathogenesis of SARS-CoV-2.

Proteolytic cleavage at the S1/S2 junction results in shedding of S1 subunit, which further exposes the spike SAg-like motif in the dissociated S1 trimer (**Figure 1C**). Interestingly, Ogata et al. detected circulating S1 in the plasma of COVID-19 patients and showed that higher concentrations of S1 correlated with COVID-19 severity (49), potentially due to increased exposure to the SARS-CoV-2 SAg-like motif. Similarly, sustained levels of circulating shed S1 are detected in the plasma of children with MIS-C, who were also found to have persistent presence of SARS-CoV-2 RNA in the gut and increased gut permeability (50).

**Figure 2:**
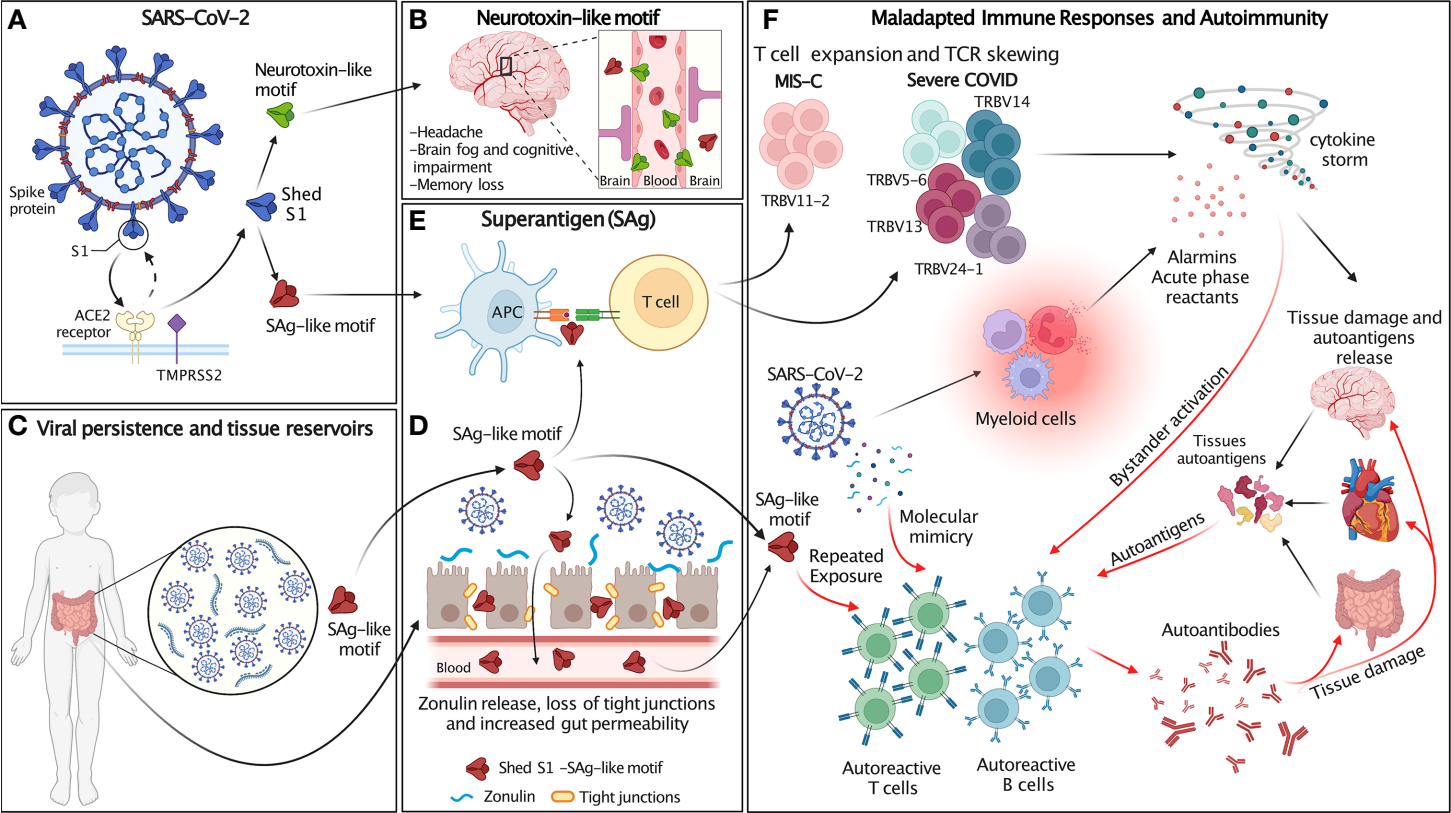
Schematic of the proposed hypothesis. **(A)** SARS-CoV-2 spike (blue) proteins expressed at the surface of SARS-CoV-2 interact with host cell ACE2 receptor (yellow) and transmembrane protease TMPRSS2 (purple). After SARS-CoV-2 spike proteins bind to ACE2, they are cleaved at the S1/S2 junction by human proteases (TMPRSS2 and furin), which mediate membrane fusion and viral cellular entry. Protease binds the spike trimer near the PRRA insert unique to SARS-CoV-2 and located in the SAg-like motif adjacent to the S1/S2 cleavage site. Cleavage of S1/S2 separates each subunit of the spike trimer into 2 subunits, S1 and S2, resulting in the S2 fusion trimer (bound to viral membrane) and the S1 trimer (released to extracellular space). The SAg and neurotoxin-like motifs are exposed in S1. **(B)** The neurotoxin-like motif in circulating SARS-CoV-2 S1 crosses the BBB and contributes to the neurological symptoms associated with MIS-C and individuals recovering from COVID infection. **(C)** SARS-CoV-2 persists in extra-pulmonary organs, including the GI tract. **(D)** Persistent presence of SARS-CoV-2 antigens in the gut results in Zonulin-mediated increased intestinal permeability and leakage of S1 and the SAg-like motif into the circulation. **(E)** The SAg-like motif in SARS-CoV-2 S1 activates a large fraction of T cell and leads to TCR skewing. **(F)** SARS-CoV-2 and the SAg-like motif in S1 triggers maladapted immune responses and autoimmunity. The SAg-like motif in shed S1 triggers T cells expansion, TCR skewing and hyperinflammation/cytokine storm, resulting in host tissue damage and autoantigen release. SARS-CoV-2 persistence in tissue reservoirs also activates autoreactive T and B cells *via* molecular mimicry. Autoreactive T and B cells may also be activated by either repeated exposure to the SAg-like motif in S1 and bystander activation. Release of autoantigens and activation of autoreactive lymphocytes leads to the production of autoantibodies that further damage host tissues. Figure created with BioRender.com.

Taken together, these findings support the hypothesis that the SAg-like motif in SARS-CoV-2 spike is a critical player in severe COVID-19 and MIS-C pathogenesis. Specifically, we speculate that by stimulating a large proportion of T cells and inducing the release of proinflammatory mediators and cytokines, this motif may act as a SAg to trigger TSS-like response in patients with severe COVID-19 and MIS-C (44, 50, 51) (**Figure 2**).

## Discovery that AN Anti-SEB Monoclonal Antibody Blocks SARS-CoV-2 Cell Entry

SEB is a bacterial toxin secreted by *Staphylococcus aureus* (*S. aureus*), which colonizes the skin and mucosal surfaces in 20 to 30% of the healthy population (52). Although *S. aureus* is ubiquitous, development of TSS after infection is a rare event, with an estimated incidence of 0.8 to 3.4 per 100,000 in the United States (53). Such low incidence may be accounted by the presence of detectable levels of antibodies against bacterial SAgs in up to 80% of individuals 12 years of age and older (54–56).

Monoclonal antibodies (mAbs) have been developed that are specific to SEB and exhibit toxin-neutralizing efficacy in murine models of TSS (57). Among these, 6D3 targets the SEB fragment that is very similar to the SARS-CoV-2 SAg-like motif (44, 58). Computational studies predict that 6D3 would bind with high affinity to the PRRA SAg-like insert in SARS-CoV-2 S1, and consequently may prevent T cell activation and cytokine storm otherwise triggered by the SAg-like motif. In addition, the antibody 6D3 was also predicted to bind to the cleavage site in spike for host cell proteases, and therefore block viral entry (58). Indeed, in an *in vitro* cell culture system, pretreatment with 6D3 significantly inhibited SARS-CoV-2 viral infection in a concentration-dependent manner (58). We note that 6D3 concentration was relatively high, which suggests that further modification of 6D3 to increase its affinity or potency might be required. The mAb 6D3 may block both the binding to the SARS-CoV2 SAg-like motif to TCR, and thus prevent inflammation, and block the S1/S2 cleavage site to reduce, if not completely inhibit, viral entry at the same time. Computational studies further predicted that the mAb 6D3 could bind to the S1 subunit of variants that exhibit mutations at the receptor binding domain of the spike, including the Omicron variant (unpublished). Therefore, humanized versions of this antibody could be of potential additional therapeutic benefit in MIS-C patients and severe COVID-19. It is also tempting to speculate that endogenous circulating anti-SEB Abs may contribute to the age-related protection against severe COVID disease; titers of antibodies against SEB are known to decrease after 70 years (59, 60), and that population is more susceptible to develop severe COVID-19. Similarly, cross-reactivity to antibodies that neutralize SEB may explain why MIS-C patients have been shown to respond well to intravenous immunoglobulin (IVIG) therapy (61, 62).

## Discovery of a Skewed Vβ TCR Repertoire in Adults With Severe COVID-19 and Children With MIS-C

Most SAgs activate T cells by cross-linking MHC class II with TCR β-chains (Vβ chains) at their variable domain (63), which results in Vβ skewing, whereby T cells with specific Vβ chains and diverse antigen specificity dominate the TCR repertoire (63). Vβ skewing with junctional diversity is, therefore, a marker of SAg involvement (44, 64). In support of our hypothesis, TCR repertoire sequencing of MIS-C patients and adult COVID-19 patients with severe hyperinflammation identified TCR Vβ skewing, characterized by the expansion of TRBV5-6, TRBV13, TRBV14, and TRBV24-1 in adults (44) and TRBV11-2 (Vβ21.3) in MIS-C patients (65). Skewing and expansion of TRBV11-2 clonotypes has been subsequently confirmed in four independent cohorts of MIS-C patients (66–69). This effect is observed in both CD4^+^ and CD8^+^ T cells, appears to be unique to MIS-C, as it is not detected in TSS or KD patients, and is transient and returns to baseline weeks after MIS-C resolution (66–69). Expanding TRBV11-2 CD4^+^ T cells also express markers of T cell activation, effector function and apoptosis (68). TRBV11-2 skewing in MIS-C patients positively correlates with disease severity and circulating levels of inflammatory markers, including TNF-α, IFN-γ, IL-6, IL-18 and IL-1RA (65, 66). Computational analysis indicates that TRBV11-2 engages in CDR3-independent interactions with the polybasic insert PRRA in the SARS-CoV-2 SAg-like motif (44, 65). Furthermore, TCR repertoire skewing, and TBRV11-2 expansion and MIS-C severity correlated with higher levels of circulating shed S1 detected in a cohort of MIS-C patients (50). However, additional functional studies are needed to confirm that TRBV11-2 clones are responsive to shed S1.

## HLA Class I Association With Severe MIS-C Children With TRBV11-2 Skewing

Conventional SAgs interact with both TCR and HLA class II molecules. Specific polymorphisms in HLA class II molecules which allow for stronger interactions with SAgs have been found to be associated with SAg-mediated diseases (70, 71). Interestingly, several studies have reported an association of HLA class I, but not class II, alleles with MIS-C. In an American cohort, a triple combination of three HLA class I alleles (A02, B35 and C04) was identified in severe MIS-C patients with TRBV11-2 expansion (4/4 MIS-C patients with TRBV11-2 expansion genotyped), but not in mild MIS-C patients without TRBV11-2 expansion (0/3 MIS-C patients without TRBV11-2 expansion genotyped) (65). In a cohort of Italian patients, 5/9 MIS-C patients possessed all three HLA class I alleles, whereas pediatric COVID-19 cases and healthy children did not, however this was independent of disease severity (68). In another American cohort, 28.6% (6/21 genotyped) of MIS-C patients possessed all three HLA class I alleles compared to none of the pediatric controls (0/7) (72). Although another study did not find such as association (66), the replication in three separate cohorts strongly indicates that possession of the three HLA class I alleles in combination confers increased susceptibility to the development of MIS-C and possibly TRBV11-2 expansion. This raises the question of whether a non-conventional SAg-like interaction involving HLA class I rather than HLA class II may occur in MIS-C. Overall, an association of HLA class I molecules with MIS-C indicates a genetic component to disease susceptibility and may explain the rarity of MIS-C and why the disease appears to disproportionally affect specific ethnic populations.

## Viral Infections and the Development of Autoimmunity

Several COVID-19 manifestations are similar to those observed in autoimmune disorders. Indeed, longitudinal immune profiling of SARS-CoV-2 infected patients indicates that severe COVID-19 is associated with overwhelming immune responses characterized by not only high levels of proinflammatory cytokines, interferons and chemokines but also the presence of autoantibodies known to target pathways involved in anti-viral responses and tissue-associated autoantigens (73–76). This is perhaps not surprising, as viral infections have previously been implicated in the development of autoimmunity. For example, the acute phase of Chikungunya viral infection is characterized by fever, rash, arthritis and joint pain, which can persist for years and appear similar to rheumatoid arthritis (RA) (77). Systemic lupus erythematosus (SLE) is an autoimmune disorder linked to infection with EBV, parvovirus B19, retroviruses and cytomegalovirus (78, 79). Similarly, EBV, which is estimated to infect 95% of the general population, was recently implicated in the development of multiple sclerosis (MS) (80). SARS-CoV-2 infection is also associated with the development of lingering post-infectious and chronic symptoms (81–84). These manifestations, also called long COVID, appear similar to ones observed during myalgic encephalomyelitis/chronic fatigue syndrome (ME/CFS), which is most commonly associated with viral infections or multiple exposures to viral and bacterial pathogens (85, 86).

Autoimmune disorders can be induced by infectious agents through the activation and clonal expansion of autoreactive lymphocytes (87–89). However, the mechanisms by which viral infections trigger the onset of autoimmunity are not completely understood. They might involve molecular mimicry, a process in which viral antigens appear similar to host self-antigens, and bystander activation, in which production of proinflammatory mediators leads to tissue damage, release of self-antigens and activation of autoreactive T and B cells (90–92).

Since SAgs are able to activate a large fraction of lymphocytes that express particular Vβ segments, including normally quiescent autoreactive T and B cells clones, and induce the release of pro-inflammatory cytokines and chemokines, SAgs may initiate autoimmunity or exacerbate already established autoinflammatory disorders (33, 93). In addition, repeated exposures to viral SAgs have been associated with the development of autoimmunity, as discussed in more detail below.

Dysregulated immune responses have been reported in patients with post-SARS-CoV-2 hyperinflammatory syndromes, including MIS-C and adults with long COVID who experience persistent symptoms weeks to months after their initial COVID-19 diagnosis (94–96). Clinical data, computational modeling and transcriptomic data suggest that the SARS-CoV-2 S1 SAg-like motif may contribute to the development of hyperinflammatory responses associated with severe COVID-19 and MIS-C. Specifically, elevated levels of circulating S1 are reported in MIS-C patients (50). S1 antigenemia correlates with a skewed TCR repertoire and expansion of TRBV11-2 clones, which is characteristic of severe MIS-C (50, 65). In addition, SARS-CoV-2 RNA and viral antigens are detected in the gut (97–99), which becomes leaky (50, 100), possibly resulting in chronic, repeated exposure to viral antigens. Indeed, in a recent study, post-acute sequelae of COVID-19 were reported by the majority of IBD patients with viral antigen persistence, but not from patients without viral antigen persistence (101).

Based on these observations, we propose that in the context of an intense antiviral immune response, inefficient viral clearance associated with SARS-CoV-2 persistence and repeated viral exposure may lead to uncontrolled and maladapted immune responses that cross-react with autoantigens. Thus, the SARS-CoV2 SAg-like motif may be involved in the development of COVID-19 hyperinflammatory syndromes, including MIS-C and autoimmune manifestations associated with long COVID. This hypothesis provides a mechanism for post-acute COVID syndromes and links these conditions with persistent presence of viral antigens, repeated exposures to viral SAgs, and subsequent development of autoimmunity (**Figure 2**).

## Immune Responses to Repeated Superantigen Exposures

In peripheral blood mononuclear cells (PBMCs) collected from individuals with TSS, *ex vivo* stimulation with toxic shock syndrome toxin-1 (TSST-1) results in the expansion of Vβ2^+^ T cells, whether patients had acute disease or were convalescent (102). Multiple episodes of menstrual TSS can occur in the same patient, with each more severe than the previous one, and such recurrence is usually associated with persistent *S. aureus* colonization and lack of toxin-neutralizing antibodies (103, 104). Recurrence of non-menstrual TSS has also been reported (105, 106). In mice, a single SEB injection induces selective activation and expansion of Vβ8^+^ T cells and massive production of cytokines (93, 107, 108). T cells isolated from SEB-pretreated mice are capable of producing IL-10 and IFN-γ upon SEB restimulation and are still functional *in vivo* since SEB-pretreated mice develop lethal shock upon SEB rechallenge (108, 109). Taken together, these observations suggest that SAg-reactive T cells are able to respond to subsequent SAg stimulations (104).

SAgs engage TCRs on T cells and bind with high affinity to MHC class II molecules, providing activation signals to MHC class II^+^ cells, including B cells (110, 111). In mice, even if repeated administration of SEB does not lead to the development of a complete autoimmune phenotype, it results in a marked hyperglobulinemia characterized by elevated levels of circulating IgG1, IgG2a and IgE, indicative of B cell activation (112). Furthermore, in mice, chronic exposure to SEB has been shown to result in systemic inflammation mimicking human SLE, which is characterized by T cell infiltration of the lungs, kidneys and liver, as well as production of autoantibodies and deposition of immune complexes in kidney glomeruli (112, 113). Thus, SAgs may initiate or accentuate autoimmune disorders by activating APCs as well as autoreactive T and B cells (33, 93).

## The Autoimmune Signatures of long COVID and MIS-C

Autoantibodies, a hallmark of autoimmune disorders, induce tissue damage and inflammation by binding to self-antigens and forming deleterious immune complexes, which further activate immune cells, deposit in tissues, and trigger the complement pathway. Autoantibodies might contribute to severe COVID-19 and long COVID (114). Multiple studies have reported elevated levels of autoantibodies in severely ill COVID-19 patients targeting nuclear antigens, phospholipids and cytokines (74, 75, 115–119). While the presence of these autoantibodies may precede COVID-19, a proportion of hospitalized COVID-19 patients develop *de novo* autoantibodies following SARS-CoV-2 infection (75).

Further studies are needed to fully understand the mechanisms leading to autoantibody induction during COVID-19 and the extent of their pathogenic role in the development of post-acute sequelae of COVID-19 (PASC), including long COVID. While most people fully recover from COVID-19, a large proportion of SARS-CoV-2 infected individuals experience at least one symptom that persists months after initial infection (81–84). Common lingering symptoms are heterogenous and may include shortness of breath, chest pain, fatigue, palpitations, arthralgia, GI symptoms, neurological and cognitive disturbances (81, 82, 84, 120, 121). COVID-19 is also associated with increased risk of developing incident diabetes during the post-acute phase of the disease (122). A longitudinal multi-omics study performed with samples collected from COVID-19 patients indicated that the presence of specific autoantibodies is associated with different PASC (83). PASC impacting the GI tract are associated with the expansion of cytotoxic T cells, including SARS-CoV-2 specific clonotypes, and bystander activation of cytomegalovirus (CMV)-specific T cells during the convalescence phase (83). These observations hint that the immune dysregulation of T and B cells and the activation of non-specific T cells associated with autoantibody production might contribute to the persistence of these long-term SARS-CoV-2 PASC.

MIS-A and MIS-C are also considered PASC, as these syndromes develop 2 to 6 weeks after known prior SARS-CoV-2 exposure or infection, and MIS-C patients are seropositive for SARS-CoV-2 S protein IgG antibodies (9–11, 14, 19, 20, 22, 123). MIS-C involves dysregulated immune responses, activation of innate and adaptive immune cells as well as elevated production of inflammatory cytokines (66, 68, 95, 123, 124). In severe MIS-C patients there is an enrichment of proteins involved in complement activation, neutrophil degranulation and dysregulated humoral responses (125). Immune profiling of MIS-C patients indicates activation of monocytes, neutrophils, and type 1 dendritic cells, decreased frequencies of circulating NK cells, and T cell lymphopenia affecting both CD4^+^ and CD8^+^ T cells, which are more proliferative and activated (95, 124, 126, 127). A subset of CD8^+^ T cells expressing the fractalkine receptor CX3CR1, and able to interact with fractalkine expressing vascular endothelium, are more activated during MIS-C (95).

Studies have reported skewed B cell responses and increased frequencies of circulating plasmablasts in adult and pediatric COVID-19 patients as well as in MIS-C (67, 69, 95, 128), particularly plasmablasts expressing the transcription factor T-bet (95). T-bet^+^ B cells accumulate with age and participate in antiviral responses (129). These cells are also detected in mice prone to developing autoimmunity and patients with autoimmune disorders and contribute to autoantibody production (130–133). Autoantibodies targeting multiple tissue self-antigens have been identified in independent cohorts of MIS-C patients and may contribute to the development of hyperinflammation (68, 69, 123, 125, 126). The 2–6-week delay between the initial SARS-CoV-2 infection and the development of MIS-C symptoms may be the period required to allow B cell activation, plasmablast induction and autoantibody production. Compared with febrile controls, MIS-C patients exhibit a significant increase in IgG autoantibodies targeting not only ubiquitously expressed antigens, but also tissue-specific antigens from the GI tract, cardiovascular and brain tissues, reflecting the systemic nature of MIS-C (125). Greater autoantibody responses were identified in a subset of patients with a hyperinflammatory blood transcriptional profile, which were also associated with disease severity and TRBV11-2 expansion (125). Furthermore, this subset of patients demonstrated strong imprints of antigenic selection in their BCR repertoires and increased usage of autoantibody associated IGHV genes (125). Neutralizing autoantibodies against interleukin-1 receptor antagonist (IL-1Ra), an anti-inflammatory protein that binds IL-1 receptor (IL-1R) and blocks IL-1α and IL-1β signaling, have been detected in a high proportion of MIS-C patients, but not in healthy controls, pediatric COVID-19 or KD samples (134). Although in that study the presence of autoantibodies against IL-1Ra did not correlate with MIS-C severity, these functional autoantibodies might exert a pathogenic role by impairing IL-1Ra bioactivity and promoting hyperinflammation during MIS-C (134). Notably, the autoantibody signatures observed in MIS-C patients cannot be explained by the history of IVIG therapy, as multiple studies have observed autoantibody enrichment prior to treatment (125, 126).

While HLA class II molecule expression is restricted to immune cells, HLA class I molecules are ubiquitously expressed by all cell types. Therefore, the presentation of SAg by HLA class I rather than class II may contribute to the multisystem inflammation that occurs in MIS-C due to widespread T cell-mediated damage of SAg-presenting cells. T-cell mediated cell damage of SAg-presenting cells may exacerbate release of damage-associated molecular pattern (DAMPS) and autoantigens, thus contributing to systemic inflammation and autoimmune responses against ubiquitously expressed intracellular autoantigens and tissue-specific autoantigens identified in MIS-C (68, 69, 123, 125, 126). For example, MHC class I presentation of SAg by endothelial cells resulting in subsequent endothelial cell damage may contribute to endothelial dysfunction and release of autoantigens observed in MIS-C. Immune profiling of MIS-C has identified enhanced activation of a subset of vascular patrolling CD8^+^ T cells (95), as well as autoantibodies directed against endothelial autoantigens (68, 69, 123, 125, 126), indicating a breakdown of immune tolerance against the endothelial compartment.

Autoantibody production to self-antigens arises from multiple factors, including the defective regulation of autoreactive B and T cells, as well as excessive antigenic drive by self-antigens released by cellular apoptosis and damaged tissue (135). Viral and bacterial infections trigger activation of autoreactive T and B cells, the transient production of autoantibodies and the development of autoimmune-like responses after the infection is cleared (133). SAgs deliver activation signals to B cells *via* MHC class II molecules expressed at their surfaces (110, 111), which may lead to inappropriate B cell responses and *de novo* production of autoantibodies, a phenotype observed in both MIS-C and in long COVID. The possibility that the SAg-like motif identified in SARS-CoV-2 S1 acts not only on T cells, but also directly on B cells to promote dysregulated B cell responses requires further studies.

## Increased Gut Permeability in MIS-C Resulting in Chronic Exposure to the Superantigen-Like Motif

MIS-C patients commonly present with severe GI symptoms, and viral RNA can be detected in their stools 2 to 6 weeks after the initial SARS-CoV-2 infection (50). Detection of autoantibodies targeting GI antigens in the plasma of MIS-C patients (123, 125) led to the hypothesis that the GI tract may serve as a persistent source of viral antigen and perhaps viral reservoirs for continuous exposure to the SARS-CoV-2 SAg-like motif. Indeed, compared with acute pediatric COVID-19, biomarkers indicating increased intestinal permeability (zonulin, LPS-binding protein, and soluble CD14) are elevated in the plasma of MIS-C patients (50).

During cellular entry, SARS-CoV-2 S1 and S2 subunits are cleaved and the trimeric S1 subunit is shed, allowing the SAg-like motif in S1 to be more exposed and accessible to interaction with immune cells (44). Sustained levels of circulating SARS-CoV-2 viral particles—including nucleocapsid, whole S and S1—are detected in the plasma of MIS-C patients weeks after the initial infection has resolved. Furthermore, a strong correlation between S1 antigenemia and TRBV11-2 skewing, which is characteristic of severe MIS-C, has been reported (50). In MIS-C patients, persistence of SARS-CoV-2 in the GI tract might promote the release of zonulin, an intestinal tight junction modulator, by intestinal epithelial cells, and lead to intestinal leakage of gut antigens and shed SARS-CoV-2 S1 into the bloodstream (50). Treatment with Larazotide (AT1001), a zonulin inhibitor capable of correcting impaired intestinal barrier, in addition to steroids and/or IVIG had favorable outcomes in a small cohort of MIS-C patients and resulted in faster resolution of GI symptoms and clearance of circulating spike antigens (50, 136). Additional studies are required to determine the full biological effect of shed S1 subunit of SARS-CoV-2 that circulates in the serum of adult COVID-19 patients (49), as well as MIS-C children (50).

## Discovery of SARS-CoV-2 Neurotoxins Predicted to Bind TCRs and Their Potential Role in Neurological Effects of COVID-19

Up to 30% of patients with MIS-C exhibit neurological symptoms, such as headache, lethargy, and confusion (13, 14, 19, 137). Neurologic manifestations are also common in adults with COVID-19 and observed in 80% of hospitalized patients (138). Loss of smell (anosmia), loss of taste (ageusia), headache, dysautonomia, neuromuscular complications, cognitive impairment or “brain fog”, memory problems, anxiety and depression are also commonly reported following COVID-19, but the mechanisms underlying these symptoms are poorly understood (120, 121). Anosmia is reported by a large fraction of COVID-19 patients. Postmortem analysis of olfactory mucosa samples from COVID-19 patients indicates that SARS-CoV-2 preferentially infects and replicates in sustentacular cells, which may indirectly affect olfactory sensory neurons (139). A longitudinal brain imaging study performed in a large cohort of UK participants scanned before and after SARS-CoV-2 infection identified impacts of COVID-19 on brain tissue structures (140). Individuals who had been infected with SARS-CoV-2 showed a consistent pattern of brain abnormalities, such as greater loss of grey matter and increased markers of tissue damage in olfactory-related brain regions (140, 141). Long COVID patients experiencing cognitive deficits or “brain fog” have increased levels of circulating CCL11, a chemokine associated with age-related cognitive decline and decreased neurogenesis (142, 143). CCL11 is also detected in the cerebrospinal fluid of SARS-CoV-2 infected mice up to 7 weeks post-infection (142). SARS-CoV-2 infection also leads to increased microglial reactivity in subcortical white matter in both mice and humans, and in mice myelin loss and depletion of oligodendrocytes has been shown to persist up to 7 weeks post-infection (142). While evidence of SARS-CoV-2 directly infecting neural tissues is scarce (144), multiple other mechanisms have been proposed to explain the effects of SARS-CoV-2 in the brain, such as blood-brain barrier (BBB) breakdown and tissue damage mediated by dysregulated host immune responses and presence of self-reactive antibodies capable of reaching the brain (145–148).

The persistent neurological symptoms reported in women recovered from TSS, including headache, cognitive impairment and memory loss (36), are remarkably similar to the neuro-psychiatric and “brain fog” symptoms described by patients suffering from long COVID. In TSS, the symptoms are believed to arise due to cytokine storm and hyperinflammation. Additionally, TSST-1 may have a direct effect on the CNS, as TSST-1 has been shown to induce cell death in neural cell cultures and can diffuse across *in vitro* models of the BBB (149).

Remarkably, the portion (Y674QTQTNSPRRAR685) of the SAg-like motif (E661-R685) identified in our recent work, which also includes the PRRA insert (44), is homologous to alpha-neurotoxin motifs from snake venom (e.g. a-cobratoxin) as well as neurotoxin-like regions from rabies virus strains (44, 150) with sequence identities of 18% to 27%. Additionally, inspired by a previous extensive analysis of superantigenic, toxin and other bioactive molecules on SARS-CoV carried out by Li and coworkers (151), we have identified three additional neurotoxin-like motifs in SARS-CoV-2 spike, including the sequence segment T299-Y351 on the S1 subunit and N777-P807 on the S2 subunit. Alignment of these two SARS-CoV-2 sequences against their counterparts in SARS-CoV showed that these motifs were highly conserved (75-84% sequence identity) between the two β-coronaviruses, and they were also predicted in computational studies to bind TCRs with high affinity (44). Notably, a recent study with a cohort not exposed to SARS-CoV-2 identified 66 epitopes with significant T cell reactivity on the SARS-CoV-2 spike (152), inviting attention to possible memory response acquired upon exposure to human common cold coronaviruses HCoV-OC43, HCoV-HKU1, HCoV-NL63, and HCoV-229E, which share sequence homology with SARS-CoV-2. The neurotoxin-like fragment T299-Y351 identified in our analysis was observed among those highly reactive epitopes, supporting the ability of this segment to bind TCRs. This suggests that this neurotoxin-like segment T299 to Y351 deserves attention as a possible source of CNS disorders observed in COVID-19 patients. Notably, this motif of 50+ amino acids spans quite a broad region on the glycoprotein, with its C-terminal portion C336-Y351 (which showed the highest reactivity in those experiments) being highly exposed and therefore prone to interactions with host cell proteins (**Figure 1E**).

The identification of these neurotoxin motifs in SARS-CoV-2 raises the possibility that spike may also have direct neurotoxic activity and contribute to the neurological symptoms associated with MIS-C and long COVID. Studies have demonstrated that the S1 subunit of SARS-CoV-2 S protein can mediate opening of the BBB (153, 154), and multiple protease cleavage sites in S1 likely results in smaller fragments (155) that may cross into the CNS. It is therefore possible that circulating S1 or S1 protein fragments may cross the BBB and directly impair neurological activity, contributing to neurological symptoms observed in MIS-C and individuals recovering from COVID infections.

## Conclusion

The observations described here, derived from computational analysis of SARS-CoV-2 sequence, structure and interactions, as well as RNA sequencing, TCR and BCR repertoire analysis, autoantibody arrays, and proteomics analysis performed on samples collected from MIS-C and COVID-19 patients, point towards a role for the SAg-like motif (residues E661-R685) we identified in SARS-CoV-2 spike in promoting hyperinflammation and potentially autoimmunity in PASC, including MIS-C and long COVID. This is of particular importance in view of a recent study showing that persistence of circulating SARS-CoV-2 Spike may be associated with post-acute COVID-19 sequelae (156). However, further studies are still required to validate our hypotheses. *In vitro* systems need to be developed to determine how chronic stimulation by SARS-CoV-2 SAg-like motif impacts T cells and B cell activation and proliferation, and to assess the neurotoxin activities of the Y674-R685 portion of this SAg-like motif as well as another segment (T299-Y351) also identified to possess neurotoxin-like properties. Improved solutions of the SARS-CoV-2 Spike structure that represent the native trimeric structure of the shed S1 subunit will be needed to conduct T and B cell stimulation experiments, as production of recombinant spike proteins usually involves deletion the reactive polybasic furin-cleavage site (PRRA), where the SAg-like motif starts. Furthermore, development of a murine *in vivo* model allowing chronic stimulation by the SAg-like motif is also needed due to the scarcity of samples from MIS-C individuals. Such a model will allow deeper mechanistic insights into MIS-C and other PASC pathogenesis, and should provide information on how chronic stimulation by the SAg-like motif impacts immune responses, as well as allow investigation of long-term sequelae and testing of potential therapeutics.

## Limitations

Since MIS-C is a novel syndrome that emerged during a world-wide pandemic, studies so far have been limited and the field lacks an established murine model. Moreover, MIS-C is a rare complication of COVID-19, so the number of patient samples available has been small, it has been challenging to achieve concomitant collection of samples from either healthy, pediatric COVID-19 or febrile control children, and it was difficult to obtain samples from patients before they have been treated, at least in the initial studies. Another limitation is the nature of biological samples collected, which are mostly peripheral blood mononuclear cells and RNA isolated from whole blood, or plasma or sera, which do not allow to investigate *in situ* muti-system immune responses. Finally, there are technical difficulties in directly investigating if the SARS-CoV2 SAg-like motif binds to TCRs and MHC class II proteins and functionally acts as a SAg by activating T and B cells in an *in vitro* system. Unlike bacterial SAgs, which are secreted toxins, the SARS-Cov-2 Spike protein, a glycosylated membrane bound trimer, exists in many conformations, and undergoes proteolytic cleavage during cell infection to release the S1 and S2 (fusion) trimers. The complex nature of the spike machinery is difficult to capture in* in vitro *systems using recombinant proteins. Preparation of recombinant viral spike protein with the endogenous sequence (PRRA) does not allow for correct folding and/or stable conformational state. Therefore, most commercially available recombinant S1 spike proteins have this key PRRA motif mutated as well as have a C-terminal His tag that is immediately proximal to the SAg-like motif, interfering with its activity. Addressing these limitations may lead to novel therapeutic approaches to MIS-C and PASC.

## Data Availability Statement

The original contributions presented in the study are included in the article/supplementary material. Further inquiries can be directed to the corresponding author.

## Author Contributions

MA conceived the idea in collaboration with IB, formulated the hypothesis, and wrote the manuscript. MNR and RP contributed to the hypothesis, wrote the manuscript and contributed to the illustrations. IB and MC performed the computational studies and analyses that led to the discovery of the SARS-CoV2-SAg-like motif and the neurotoxin motifs, and contributed to the illustrations. All authors contributed to writing the article and approved the final submitted version.

## Funding

Research was supported by the NIH grants R01AI072726 and R01AI072726-10S to MA, and P41 GM103712 and R01 GM139297 to IB. MNR is supported by the NIH grants R01HL139766 and R01HL159297, RP is supported by the American Heart Association Career Development Award (AHA 20CDA35260258) and the Cedars-Sinai Precision Health Award.

## Conflict of Interest

The authors declare that the research was conducted in the absence of any commercial or financial relationships that could be construed as a potential conflict of interest.

## Publisher’s Note

All claims expressed in this article are solely those of the authors and do not necessarily represent those of their affiliated organizations, or those of the publisher, the editors and the reviewers. Any product that may be evaluated in this article, or claim that may be made by its manufacturer, is not guaranteed or endorsed by the publisher.

## References

[1] Grasselli G Tonetti T Protti A Langer T Girardis M Bellani G. Pathophysiology of Covid-19-Associated Acute Respiratory Distress Syndrome: A Multicentre Prospective Observational Study. Lancet Respir Med (2020) 8(12):1201–8. doi: 10.1016/s2213-2600(20)30370-2 PMC7834127 32861276

[2] Wiersinga WJ Rhodes A Cheng AC Peacock SJ Prescott HC. Pathophysiology, Transmission, Diagnosis, and Treatment of Coronavirus Disease 2019 (Covid-19): A Review. JAMA (2020) 324(8):782–93. doi: 10.1001/jama.2020.12839 32648899

[3] Zhou F Yu T Du R Fan G Liu Y Liu Z. Clinical Course and Risk Factors for Mortality of Adult Inpatients With Covid-19 in Wuhan, China: A Retrospective Cohort Study. Lancet (2020) 395(10229):1054–62. doi: 10.1016/s0140-6736(20)30566-3 PMC7270627 32171076

[4] Gao YD Ding M Dong X Zhang JJ Kursat Azkur A Azkur D. Risk Factors for Severe and Critically Ill Covid-19 Patients: A Review. Allergy (2021) 76(2):428–55. doi: 10.1111/all.14657 33185910

[5] Booth A Reed AB Ponzo S Yassaee A Aral M Plans D. Population Risk Factors for Severe Disease and Mortality in Covid-19: A Global Systematic Review and Meta-Analysis. PLoS One (2021) 16(3):e0247461. doi: 10.1371/journal.pone.0247461 33661992 PMC7932512

[6] Chou J Thomas PG Randolph AG. Immunology of Sars-Cov-2 Infection in Children. Nat Immunol (2022) 23(2):177–85. doi: 10.1038/s41590-021-01123-9 PMC8981222 35105983

[7] Dowell AC Butler MS Jinks E Tut G Lancaster T Sylla P. Children Develop Robust and Sustained Cross-Reactive Spike-Specific Immune Responses to Sars-Cov-2 Infection. Nat Immunol (2022) 23(1):40–9. doi: 10.1038/s41590-021-01089-8 PMC8709786 34937928

[8] Brodin P. Sars-Cov-2 Infections in Children: Understanding Diverse Outcomes. Immunity (2022) 55(2):201–9. doi: 10.1016/j.immuni.2022.01.014 PMC8769938 35093190

[9] Verdoni L Mazza A Gervasoni A Martelli L Ruggeri M Ciuffreda M. An Outbreak of Severe Kawasaki-Like Disease at the Italian Epicentre of the Sars-Cov-2 Epidemic: An Observational Cohort Study. Lancet (2020) 395(10239):1771–8. doi: 10.1016/s0140-6736(20)31103-x PMC7220177 32410760

[10] Riphagen S Gomez X Gonzalez-Martinez C Wilkinson N Theocharis P. Hyperinflammatory Shock in Children During Covid-19 Pandemic. Lancet (2020) 395(10237):1607–8. doi: 10.1016/s0140-6736(20)31094-1 PMC7204765 32386565

[11] Whittaker E Bamford A Kenny J Kaforou M Jones CE Shah P. Clinical Characteristics of 58 Children With a Pediatric Inflammatory Multisystem Syndrome Temporally Associated With Sars-Cov-2. Jama (2020) 324(3):259–69. doi: 10.1001/jama.2020.10369 PMC7281356 32511692

[12] Feldstein LR Rose EB Horwitz SM Collins JP Newhams MM Son MBF. Multisystem Inflammatory Syndrome in U.S. Children and Adolescents. New Engl J Med (2020) 383(4):334–46 . doi: 10.1056/nejmoa2021680 PMC7346765 32598831

[13] Dufort EM Koumans EH Chow EJ Rosenthal EM Muse A Rowlands J. Multisystem Inflammatory Syndrome in Children in New York State. New Engl J Med (2020) 383(4):347–58. doi: 10.1056/nejmoa2021756 PMC7346766 32598830

[14] Belhadjer Z Méot M Bajolle F Khraiche D Legendre A Abakka S. Acute Heart Failure in Multisystem Inflammatory Syndrome in Children in the Context of Global Sars-Cov-2 Pandemic. Circulation (2020) 142(5):429–36. doi: 10.1161/circulationaha.120.048360 32418446

[15] Shulman ST. Pediatric Coronavirus Disease-2019-Associated Multisystem Inflammatory Syndrome. J Pediatr Infect Dis Soc (2020) 9(3):285–6. doi: 10.1093/jpids/piaa062 PMC7313948 32441751

[16] Rowley AH. Understanding Sars-Cov-2-Related Multisystem Inflammatory Syndrome in Children. Nat Rev Immunol (2020), 20(8):453–4 . doi: 10.1038/s41577-020-0367-5 PMC7296515 32546853

[17] Vella LA Rowley AH. Current Insights Into the Pathophysiology of Multisystem Inflammatory Syndrome in Children. Curr Pediatr Rep (2021), 1–10 Epub 20211019. doi: 10.1007/s40124-021-00257-6 34692237 PMC8524214

[18] Morris SB Schwartz NG Patel P Abbo L Beauchamps L Balan S. Case Series of Multisystem Inflammatory Syndrome in Adults Associated With Sars-Cov-2 Infection - United Kingdom and United States, March-August 2020. MMWR Morb Mortal Wkly Rep (2020) 69(40):1450–6. doi: 10.15585/mmwr.mm6940e1 PMC7561225 33031361

[19] Toubiana J Poirault C Corsia A Bajolle F Fourgeaud J Angoulvant F. Kawasaki-Like Multisystem Inflammatory Syndrome in Children During the Covid-19 Pandemic in Paris, France: Prospective Observational Study. Bmj (2020) 369:m2094. doi: 10.1136/bmj.m2094 32493739 PMC7500538

[20] Cheung EW Zachariah P Gorelik M Boneparth A Kernie SG Orange JS. Multisystem Inflammatory Syndrome Related to Covid-19 in Previously Healthy Children and Adolescents in New York City. Jama (2020) 324(3):294–6. doi: 10.1001/jama.2020.10374 PMC7281352 32511676

[21] CDC. Health Department-Reported Cases of Multisystem Inflammatory Syndrome in Children (Mis-C) in the United States (2022). Available at: https://covidcdcgov/covid-data-tracker/#mis-national-surveillance.

[22] Belay ED Abrams J Oster ME Giovanni J Pierce T Meng L. Trends in Geographic and Temporal Distribution of Us Children With Multisystem Inflammatory Syndrome During the Covid-19 Pandemic. JAMA Pediatr (2021) 175(8):837–45. doi: 10.1001/jamapediatrics.2021.0630 PMC8025123 33821923

[23] McCrindle BW Rowley AH Newburger JW Burns JC Bolger AF Gewitz M. Diagnosis, Treatment, and Long-Term Management of Kawasaki Disease: A Scientific Statement for Health Professionals From the American Heart Association. Circulation (2017) 135(17):e927–e99. doi: 10.1161/cir.0000000000000484 28356445

[24] Soni PR Noval Rivas M Arditi M. A Comprehensive Update on Kawasaki Disease Vasculitis and Myocarditis. Curr Rheumatol Rep (2020) 22(2):6. doi: 10.1007/s11926-020-0882-1 32020498

[25] Newburger JW Takahashi M Burns JC. Kawasaki Disease. J Am Coll Cardiol (2016) 67(14):1738–49. doi: 10.1016/j.jacc.2015.12.073 27056781

[26] Godfred-Cato S Abrams JY Balachandran N Jaggi P Jones K Rostad CA. Distinguishing Multisystem Inflammatory Syndrome in Children From Covid-19, Kawasaki Disease and Toxic Shock Syndrome. Pediatr Infect Dis J (2022) 41(4):315–23. doi: 10.1097/inf.0000000000003449 PMC8919949 35093995

[27] Farooqi KM Chan A Weller RJ Mi J Jiang P Abrahams E. Longitudinal Outcomes for Multisystem Inflammatory Syndrome in Children. Pediatrics (2021) 148(2):e2021051155. doi: 10.1542/peds.2021-051155 34266903

[28] Jhaveri S Ahluwalia N Kaushik S Trachtman R Kowalsky S Aydin S. Longitudinal Echocardiographic Assessment of Coronary Arteries and Left Ventricular Function Following Multisystem Inflammatory Syndrome in Children. J Pediatr (2021) 228:290–3.e1. doi: 10.1016/j.jpeds.2020.08.002 PMC7403848 32768467

[29] Cook A Janse S Watson JR Erdem G. Manifestations of Toxic Shock Syndrome in Children, Columbus, Ohio, USA, 2010-2017(1). Emerg Infect Dis (2020) 26(6):1077–83. doi: 10.3201/eid2606.190783 PMC7258457 32442091

[30] Low DE. Toxic Shock Syndrome: Major Advances in Pathogenesis, But Not Treatment. Crit Care Clin (2013) 29(3):651–75. doi: 10.1016/j.ccc.2013.03.012 23830657

[31] Basalely A Gurusinghe S Schneider J Shah SS Siegel LB Pollack G. Acute Kidney Injury in Pediatric Patients Hospitalized With Acute Covid-19 and Multisystem Inflammatory Syndrome in Children Associated With Covid-19. Kidney Int (2021) 100(1):138–45. doi: 10.1016/j.kint.2021.02.026 PMC7927648 33675848

[32] Krakauer T. Staphylococcal Superantigens: Pyrogenic Toxins Induce Toxic Shock. Toxins (2019) 11(3):178. doi: 10.3390/toxins11030178 PMC6468478 30909619

[33] Chatila T Geha RS. Superantigens. Curr Opin Immunol (1992) 4(1):74–8. doi: 10.1016/0952-7915(92)90129-3 1596371

[34] Chesney PJ Bergdoll MS Davis JP Vergeront JM. The Disease Spectrum, Epidemiology, and Etiology of Toxic-Shock Syndrome. Annu Rev Microbiol (1984) 38:315–38. doi: 10.1146/annurev.mi.38.100184.001531 6388495

[35] Todd JK. Toxic Shock Syndrome. Clin Microbiol Rev (1988) 1(4):432–46. doi: 10.1128/cmr.1.4.432 PMC358064 3069202

[36] Rosene KA Copass MK Kastner LS Nolan CM Eschenbach DA. Persistent Neuropsychological Sequelae of Toxic Shock Syndrome. Ann Intern Med (1982) 96(6 Pt 2):865–70. doi: 10.7326/0003-4819-96-6-865 7091958

[37] Sutkowski N Palkama T Ciurli C Sekaly RP Thorley-Lawson DA Huber BT. An Epstein-Barr Virus-Associated Superantigen. J Exp Med (1996) 184(3):971–80. doi: 10.1084/jem.184.3.971 PMC2192769 9064357

[38] Raftery MJ Behrens CK Müller A Krammer PH Walczak H Schönrich G. Herpes Simplex Virus Type 1 Infection of Activated Cytotoxic T Cells: Induction of Fratricide as a Mechanism of Viral Immune Evasion. J Exp Med (1999) 190(8):1103–14. doi: 10.1084/jem.190.8.1103 PMC2195666 10523608

[39] Leroy EM Becquart P Wauquier N Baize S. Evidence for Ebola Virus Superantigen Activity. J Virol (2011) 85(8):4041–2. doi: 10.1128/jvi.00181-11 PMC3126126 21307193

[40] Conrad B Weissmahr RN Böni J Arcari R Schüpbach J Mach B. A Human Endogenous Retroviral Superantigen as Candidate Autoimmune Gene in Type I Diabetes. Cell (1997) 90(2):303–13. doi: 10.1016/s0092-8674(00)80338-4 9244304

[41] Dobrescu D Ursea B Pope M Asch AS Posnett DN. Enhanced Hiv-1 Replication in V Beta 12 T Cells Due to Human Cytomegalovirus in Monocytes: Evidence for a Putative Herpesvirus Superantigen. Cell (1995) 82(5):753–63. doi: 10.1016/0092-8674(95)90472-7 7671303

[42] Karray S Zouali M. Identification of the B Cell Superantigen-Binding Site of Hiv-1 Gp120. Proc Natl Acad Sci (1997) 94(4):1356–60. doi: 10.1073/pnas.94.4.1356 PMC19795 9037057

[43] Lafon M Lafage M Martinez-Arends A Ramirez R Vuillier F Charron D. Evidence for a Viral Superantigen in Humans. Nature (1992) 358(6386):507–10. doi: 10.1038/358507a0 1386410

[44] Cheng MH Zhang S Porritt RA Noval Rivas M Paschold L Willscher E. Superantigenic Character of an Insert Unique to Sars-Cov-2 Spike Supported by Skewed Tcr Repertoire in Patients With Hyperinflammation. Proc Natl Acad Sci U.S.A. (2020) 117(41):25254–62. doi: 10.1073/pnas.2010722117 PMC7568239 32989130

[45] Walls AC Park Y-J Tortorici MA Wall A McGuire AT Veesler D. Structure, Function, and Antigenicity of the Sars-Cov-2 Spike Glycoprotein. Cell (2020) 181(2):281–92.e6. doi: 10.1016/j.cell.2020.02.058 32155444 PMC7102599

[46] Jackson CB Farzan M Chen B Choe H. Mechanisms of Sars-Cov-2 Entry Into Cells. Nat Rev Mol Cell Biol (2022) 23(1):3–20. doi: 10.1038/s41580-021-00418-x 34611326 PMC8491763

[47] Johnson BA Xie X Bailey AL Kalveram B Lokugamage KG Muruato A. Loss of Furin Cleavage Site Attenuates Sars-Cov-2 Pathogenesis. Nature (2021) 591(7849):293–9. doi: 10.1038/s41586-021-03237-4 PMC8175039 33494095

[48] Lau S-Y Wang P Mok BW-Y Zhang AJ Chu H Lee AC-Y. Attenuated Sars-Cov-2 Variants With Deletions at the S1/S2 Junction. Emerging Microbes Infections (2020) 9(1):837–42. doi: 10.1080/22221751.2020.1756700 PMC7241555 32301390

[49] Ogata AF Maley AM Wu C Gilboa T Norman M Lazarovits R. Ultra-Sensitive Serial Profiling of Sars-Cov-2 Antigens and Antibodies in Plasma to Understand Disease Progression in Covid-19 Patients With Severe Disease. Clin Chem (2020) 66(12):1562–72. doi: 10.1093/clinchem/hvaa213 PMC7499543 32897389

[50] Yonker LM Gilboa T Ogata AF Senussi Y Lazarovits R Boribong BP. Multisystem Inflammatory Syndrome in Children Is Driven by Zonulin-Dependent Loss of Gut Mucosal Barrier. J Clin Invest (2021) 131(14):e149633. doi: 10.1172/jci149633 PMC8279585 34032635

[51] Noval Rivas M Porritt RA Cheng MH Bahar I Arditi M. Covid-19-Associated Multisystem Inflammatory Syndrome in Children (Mis-C): A Novel Disease That Mimics Toxic Shock Syndrome-The Superantigen Hypothesis. J Allergy Clin Immunol (2021) 147(1):57–9. doi: 10.1016/j.jaci.2020.10.008 PMC7564502 33075409

[52] Graham PL 3rd Lin SX Larson EL. A U.S. Population-Based Survey of Staphylococcus Aureus Colonization. Ann Intern Med (2006) 144(5):318–25. doi: 10.7326/0003-4819-144-5-200603070-00006 16520472

[53] Ross A Shoff HW. Toxic Shock Syndrome. In: Statpearls. Treasure Island (FL) StatPearls Publishing LLC (2022). Available from: https://www.ncbi.nlm.nih.gov/books/NBK459345/?report=classic 29083727

[54] LeClaire RD Bavari S. Human Antibodies to Bacterial Superantigens and Their Ability to Inhibit T-Cell Activation and Lethality. Antimicrob Agents Chemother (2001) 45(2):460–3. doi: 10.1128/aac.45.2.460-463.2001 PMC90313 11158741

[55] McGann VG Rollins JB Mason DW. Evaluation of Resistance to Staphylococcal Enterotoxin B: Naturally Acquired Antibodies of Man and Monkey. J Infect Dis (1971) 124(2):206–13. doi: 10.1093/infdis/124.2.206 5001454

[56] Campbell DE Kemp AS. Production of Antibodies to Staphylococcal Superantigens in Atopic Dermatitis. Arch Dis Child (1998) 79(5):400–4. doi: 10.1136/adc.79.5.400 PMC1717757 10193251

[57] Varshney AK Wang X Cook E Dutta K Scharff MD Goger MJ. Generation, Characterization, and Epitope Mapping of Neutralizing and Protective Monoclonal Antibodies Against Staphylococcal Enterotoxin B-Induced Lethal Shock *<Sup></Sup>. J Biol Chem (2011) 286(11):9737–47. doi: 10.1074/jbc.M110.212407 PMC3059042 21233204

[58] Cheng MH Porritt RA Rivas MN Krieger JM Ozdemir AB Garcia G Jr.. A Monoclonal Antibody Against Staphylococcal Enterotoxin B Superantigen Inhibits Sars-Cov-2 Entry *In Vitro* . Structure (2021) 29(9):951–62.e3. doi: 10.1016/j.str.2021.04.005 33930306 PMC8082696

[59] Meyer TC Michalik S Holtfreter S Weiss S Friedrich N Völzke H. A Comprehensive View on the Human Antibody Repertoire Against Staphylococcus Aureus Antigens in the General Population. Front Immunol (2021) 12:651619. doi: 10.3389/fimmu.2021.651619 33777051 PMC7987813

[60] Vergeront JM Stolz SJ Crass BA Nelson DB Davis JP Bergdoll MS. Prevalence of Serum Antibody to Staphylococcal Enterotoxin F Among Wisconsin Residents: Implications for Toxic-Shock Syndrome. J Infect Dis (1983) 148(4):692–8. doi: 10.1093/infdis/148.4.692 6631061

[61] Takei S Arora YK Walker SM. Intravenous Immunoglobulin Contains Specific Antibodies Inhibitory to Activation of T Cells by Staphylococcal Toxin Superantigens [See Comment]. J Clin Invest (1993) 91(2):602–7. doi: 10.1172/JCI116240 PMC287991 8432865

[62] Nishi JI Kanekura S Takei S Kitajima I Nakajima T Wahid MR. B Cell Epitope Mapping of the Bacterial Superantigen Staphylococcal Enterotoxin B: The Dominant Epitope Region Recognized by Intravenous Igg. J Immunol (1997) 158(1):247–54. 8977196

[63] Deacy AM Gan SK-E Derrick JP. Superantigen Recognition and Interactions: Functions, Mechanisms and Applications. Front Immunol (2021) 12:731845. doi: 10.3389/fimmu.2021.731845 34616400 PMC8488440

[64] Hongmin Li Llera A Malchiodi EL Mariuzza RA. The Structural Basis of T Cell Activation by Superantigens. Annu Rev Immunol (1999) 17(1):435–66. doi: 10.1146/annurev.immunol.17.1.435 10358765

[65] Porritt RA Paschold L Rivas MN Cheng MH Yonker LM Chandnani H. HLA Class I-Associated Expansion of Trbv11-2 T Cells in Multisystem Inflammatory Syndrome in Children. J Clin Invest (2021) 131(10):e146614. doi: 10.1172/jci146614 PMC8121516 33705359

[66] Moreews M Le Gouge K Khaldi-Plassart S Pescarmona R Mathieu AL Malcus C. Polyclonal Expansion of Tcr Vbeta 21.3(+) Cd4(+) and Cd8(+) T Cells Is a Hallmark of Multisystem Inflammatory Syndrome in Children. Sci Immunol (2021) 6(59):eabh1516. doi: 10.1126/sciimmunol.abh1516 34035116 PMC8815705

[67] Hoste L Roels L Naesens L Bosteels V Vanhee S Dupont S. Tim3+ Trbv11-2 T Cells and Ifngamma Signature in Patrolling Monocytes and Cd16+ Nk Cells Delineate Mis-C. J Exp Med (2022) 219(2):e20211381. doi: 10.1084/jem.20211381 34914824 PMC8685281

[68] Sacco K Castagnoli R Vakkilainen S Liu C Delmonte OM Oguz C. Immunopathological Signatures in Multisystem Inflammatory Syndrome in Children and Pediatric Covid-19. Nat Med (2022) 28(5):1050–62. doi: 10.1038/s41591-022-01724-3 PMC9119950 35177862

[69] Ramaswamy A Brodsky NN Sumida TS Comi M Asashima H Hoehn KB. Immune Dysregulation and Autoreactivity Correlate With Disease Severity in Sars-Cov-2-Associated Multisystem Inflammatory Syndrome in Children. Immunity (2021) 54(5):1083–95.e7. doi: 10.1016/j.immuni.2021.04.003 33891889 PMC8043654

[70] Llewelyn M Sriskandan S Peakman M Ambrozak DR Douek DC Kwok WW. Hla Class Ii Polymorphisms Determine Responses to Bacterial Superantigens. J Immunol (2004) 172(3):1719–26. doi: 10.4049/jimmunol.172.3.1719 14734754

[71] Llewelyn M. Human Leukocyte Antigen Class Ii Haplotypes That Protect Against or Predispose to Streptococcal Toxic Shock. Clin Infect Dis (2005) 41(Supplement_7):S445–S8. doi: 10.1086/431986 16237645

[72] Conway SR Lazarski CA Field NE Jensen-Wachspress M Lang H Kankate V. Sars-Cov-2-Specific T Cell Responses Are Stronger in Children With Multisystem Inflammatory Syndrome Compared to Children With Uncomplicated Sars-Cov-2 Infection. Front Immunol (2022) 12:793197. doi: 10.3389/fimmu.2021.793197 35116027 PMC8803660

[73] Lucas C Wong P Klein J Castro TBR Silva J Sundaram M. Longitudinal Analyses Reveal Immunological Misfiring in Severe Covid-19. Nature (2020) 584(7821):463–9. doi: 10.1038/s41586-020-2588-y PMC7477538 32717743

[74] Wang EY Mao T Klein J Dai Y Huck JD Jaycox JR. Diverse Functional Autoantibodies in Patients With Covid-19. Nature (2021) 595(7866):283–8. doi: 10.1038/s41586-021-03631-y PMC13130511 34010947

[75] Chang SE Feng A Meng W Apostolidis SA Mack E Artandi M. New-Onset Igg Autoantibodies in Hospitalized Patients With Covid-19. Nat Commun (2021) 12(1):5417. doi: 10.1038/s41467-021-25509-3 34521836 PMC8440763

[76] Bhadelia N Belkina AC Olson A Winters T Urick P Lin N. Distinct Autoimmune Antibody Signatures Between Hospitalized Acute Covid-19 Patients, Sars-Cov-2 Convalescent Individuals, and Unexposed Pre-Pandemic Controls. Medrxiv (2021) 2021.01.21.21249176. doi: 10.1101/2021.01.21.21249176

[77] Tanay A. Chikungunya Virus and Autoimmunity. Curr Opin Rheumatol (2017) 29(4):389–93. doi: 10.1097/bor.0000000000000396 28376065

[78] Jog NR James JA. Epstein Barr Virus and Autoimmune Responses in Systemic Lupus Erythematosus. Front Immunol (2021) 11:623944. doi: 10.3389/fimmu.2020.623944 33613559 PMC7886683

[79] Ramos-Casals M. Viruses and Lupus: The Viral Hypothesis. LUPUS (2008) 17(3):163–5. doi: 10.1177/0961203307086268 18372354

[80] Bjornevik K Cortese M Healy BC Kuhle J Mina MJ Leng Y. Longitudinal Analysis Reveals High Prevalence of Epstein-Barr Virus Associated With Multiple Sclerosis. Science (2022) 375(6578):296–301. doi: 10.1126/science.abj8222 35025605

[81] Nalbandian A Sehgal K Gupta A Madhavan MV McGroder C Stevens JS. Post-Acute Covid-19 Syndrome. Nat Med (2021) 27(4):601–15. doi: 10.1038/s41591-021-01283-z PMC8893149 33753937

[82] Sudre CH Murray B Varsavsky T Graham MS Penfold RS Bowyer RC. Attributes and Predictors of Long Covid. Nat Med (2021) 27(4):626–31. doi: 10.1038/s41591-021-01292-y PMC7611399 33692530

[83] Su Y Yuan D Chen DG Ng RH Wang K Choi J. Multiple Early Factors Anticipate Post-Acute Covid-19 Sequelae. Cell (2022) 185(5):881–95.e20. doi: 10.1016/j.cell.2022.01.014 35216672 PMC8786632

[84] Groff D Sun A Ssentongo AE Ba DM Parsons N Poudel GR. Short-Term and Long-Term Rates of Postacute Sequelae of Sars-Cov-2 Infection: A Systematic Review. JAMA Network Open (2021) 4(10):e2128568–e. doi: 10.1001/jamanetworkopen.2021.28568 PMC8515212 34643720

[85] Komaroff AL Bateman L. Will Covid-19 Lead to Myalgic Encephalomyelitis/Chronic Fatigue Syndrome? Front Med (2021) 7:606824. doi: 10.3389/fmed.2020.606824 PMC7848220 33537329

[86] Choutka J Jansari V Hornig M Iwasaki A. Unexplained Post-Acute Infection Syndromes. Nat Med (2022) 28(5):911–23. doi: 10.1038/s41591-022-01810-6 35585196

[87] Wucherpfennig KW. Mechanisms for the Induction of Autoimmunity by Infectious Agents. J Clin Invest (2001) 108(8):1097–104. doi: 10.1172/JCI14235 PMC209539 11602615

[88] Getts DR Chastain EM Terry RL Miller SD. Virus Infection, Antiviral Immunity, and Autoimmunity. Immunol Rev (2013) 255(1):197–209. doi: 10.1111/imr.12091 23947356 PMC3971377

[89] Fujinami RS. Viruses and Autoimmune Disease - Two Sides of the Same Coin? Trends Microbiol (2001) 9(8):377–81. doi: 10.1016/S0966-842X(01)02097-2 PMC7127302 11514220

[90] Rojas M Restrepo-Jiménez P Monsalve DM Pacheco Y Acosta-Ampudia Y Ramírez-Santana C. Molecular Mimicry and Autoimmunity. J Autoimmun (2018) 95:100–23. doi: 10.1016/j.jaut.2018.10.012 30509385

[91] Smatti MK Cyprian FS Nasrallah GK Al Thani AA Almishal RO Yassine HM. Viruses and Autoimmunity: A Review on the Potential Interaction and Molecular Mechanisms. Viruses (2019) 11(8):762. doi: 10.3390/v11080762 PMC6723519 31430946

[92] Münz C Lünemann JD Getts MT Miller SD. Antiviral Immune Responses: Triggers of or Triggered by Autoimmunity? Nat Rev Immunol (2009) 9(4):246–58. doi: 10.1038/nri2527 PMC2854652 19319143

[93] Krakauer T Pradhan K Stiles BG. Staphylococcal Superantigens Spark Host-Mediated Danger Signals. Front Immunol (2016) 7:23. doi: 10.3389/fimmu.2016.00023 26870039 PMC4735405

[94] Kuri-Cervantes L Pampena MB Meng W Rosenfeld AM Ittner CAG Weisman AR. Comprehensive Mapping of Immune Perturbations Associated With Severe Covid-19. Sci Immunol (2020) 5(49) :eabd7114. doi: 10.1126/sciimmunol.abd7114 32669287 PMC7402634

[95] Vella LA Giles JR Baxter AE Oldridge DA Diorio C Kuri-Cervantes L. Deep Immune Profiling of Mis-C Demonstrates Marked But Transient Immune Activation Compared to Adult and Pediatric Covid-19. Sci Immunol (2021) 6(57):eabf7570. doi: 10.1126/sciimmunol.abf7570 33653907 PMC8128303

[96] Phetsouphanh C Darley DR Wilson DB Howe A Munier CML Patel SK. Immunological Dysfunction Persists for 8 Months Following Initial Mild-To-Moderate Sars-Cov-2 Infection. Nat Immunol (2022) 23(2):210–6. doi: 10.1038/s41590-021-01113-x 35027728

[97] Natarajan A Zlitni S Brooks EF Vance SE Dahlen A Hedlin H. Gastrointestinal Symptoms and Fecal Shedding of Sars-Cov-2 Rna Suggest Prolonged Gastrointestinal Infection. Med. (2022) 3(6):371–87.e9. doi. doi: 10.1016/j.medj.2022.04.001 PMC9005383 35434682

[98] Gaebler C Wang Z Lorenzi JCC Muecksch F Finkin S Tokuyama M. Evolution of Antibody Immunity to Sars-Cov-2. Nature (2021) 591(7851):639–44. doi: 10.1038/s41586-021-03207-w PMC8221082 33461210

[99] Cheung CCL Goh D Lim X Tien TZ Lim JCT Lee JN. Residual Sars-Cov-2 Viral Antigens Detected in Gi and Hepatic Tissues From Five Recovered Patients With Covid-19. Gut (2022) 71(1):226–9. doi: 10.1136/gutjnl-2021-324280 34083386

[100] Giron LB Dweep H Yin X Wang H Damra M Goldman AR. Plasma Markers of Disrupted Gut Permeability in Severe Covid-19 Patients. Front Immunol (2021) 12:686240. doi: 10.3389/fimmu.2021.686240 34177935 PMC8219958

[101] Zollner A Koch R Jukic A Pfister A Meyer M Rössler A. Post-Acute Covid-19 Is Characterized by Gut Viral Antigen Persistence in Inflammatory Bowel Diseases. Gastroenterology (2022) S0016-5085(22):00450-4. doi: 10.1053/j.gastro.2022.04.037 PMC9057012 35508284

[102] Rasigade JP Thomas D Perpoint T Peyramond D Chidiac C Etienne J. T-Cell Response to Superantigen Restimulation During Menstrual Toxic Shock Syndrome. FEMS Immunol Med Microbiol (2011) 62(3):368–71. doi: 10.1111/j.1574-695X.2011.00808.x 21492259

[103] Davis JP Chesney PJ Wand PJ LaVenture M. Toxic-Shock Syndrome: Epidemiologic Features, Recurrence, Risk Factors, and Prevention. N Engl J Med (1980) 303(25):1429–35. doi: 10.1056/nejm198012183032501 7432401

[104] Xu SX McCormick JK. Staphylococcal Superantigens in Colonization and Disease. Front Cell Infect Microbiol (2012) 2:52. doi: 10.3389/fcimb.2012.00052 22919643 PMC3417409

[105] Kain KC Schulzer M Chow AW. Clinical Spectrum of Nonmenstrual Toxic Shock Syndrome (Tss): Comparison With Menstrual Tss by Multivariate Discriminant Analyses. Clin Infect Dis (1993) 16(1):100–6. doi: 10.1093/clinids/16.1.100 8448283

[106] Andrews MM Parent EM Barry M Parsonnet J. Recurrent Nonmenstrual Toxic Shock Syndrome: Clinical Manifestations, Diagnosis, and Treatment. Clin Infect Dis (2001) 32(10):1470–9. doi: 10.1086/320170 11317249

[107] MacDonald HR Lees RK Baschieri S Herrmann T Lussow AR. Peripheral T-Cell Reactivity to Bacterial Superantigens *in Vivo*: The Response/Anergy Paradox. Immunol Rev (1993) 133:105–17. doi: 10.1111/j.1600-065x.1993.tb01512.x 8225363

[108] Florquin S Amraoui Z Goldman M. T Cells Made Deficient in Interleukin-2 Production by Exposure to Staphylococcal Enterotoxin B *in Vivo* Are Primed for Interferon-Gamma and Interleukin-10 Secretion. Eur J Immunol (1995) 25(5):1148–53. doi: 10.1002/eji.1830250503 7774618

[109] Gaus H Miethke T Wagner H Heeg K. Superantigen-Induced Anergy of V Beta 8+ Cd4+ T Cells Induces Functional But Non-Proliferative T Cells *in Vivo* . Immunology (1994) 83(3):333–40. PMC1415051 7835956

[110] Mourad W Al-Daccak R Chatila T Geha RS. Staphylococcal Superantigens as Inducers of Signal Transduction in MHC Class II-Positive Cells. Semin Immunol (1993) 5(1):47–55. doi: 10.1006/smim.1993.1007 8467095

[111] Scholl PR Geha RS. MHC Class II Signaling in B-Cell Activation. Immunol Today (1994) 15(9):418–22. doi: 10.1016/0167-5699(94)90271-2 7524519

[112] Florquin S Amraoui Z Goldman M. Persistent Production of Th2-Type Cytokines and Polyclonal B Cell Activation After Chronic Administration of Staphylococcal Enterotoxin B in Mice. J Autoimmun (1996) 9(5):609–15. doi: 10.1006/jaut.1996.0080 8933276

[113] Chowdhary VR Tilahun AY Clark CR Grande JP Rajagopalan G. Chronic Exposure to Staphylococcal Superantigen Elicits a Systemic Inflammatory Disease Mimicking Lupus. J Immunol (2012) 189(4):2054–62. doi: 10.4049/jimmunol.1201097 PMC3462343 22798666

[114] Khamsi R. Rogue Antibodies Could Be Driving Severe Covid-19. Nature (2021) 590(7844):29–31. doi: 10.1038/d41586-021-00149-1 33469204

[115] Bastard P Rosen LB Zhang Q Michailidis E Hoffmann HH Zhang Y. Autoantibodies Against Type I Ifns in Patients With Life-Threatening Covid-19. Science (2020) 370(6515):eabd4585. doi: 10.1126/science.abd4585 32972996 PMC7857397

[116] Vlachoyiannopoulos PG Magira E Alexopoulos H Jahaj E Theophilopoulou K Kotanidou A. Autoantibodies Related to Systemic Autoimmune Rheumatic Diseases in Severely Ill Patients With Covid-19. Ann Rheum Dis (2020) 79(12):1661–3. doi: 10.1136/annrheumdis-2020-218009 32581086

[117] Liu Y Ebinger JE Mostafa R Budde P Gajewski J Walker B. Paradoxical Sex-Specific Patterns of Autoantibody Response to Sars-Cov-2 Infection. J Transl Med (2021) 19(1):524. doi: 10.1186/s12967-021-03184-8 34965855 PMC8716184

[118] Zuo Y Estes SK Ali RA Gandhi AA Yalavarthi S Shi H. Prothrombotic Autoantibodies in Serum From Patients Hospitalized With Covid-19. Sci Trans Med (2020) 12(570):eabd3876. doi: 10.1126/scitranslmed.abd3876 PMC7724273 33139519

[119] Woodruff MC Ramonell RP Saini AS Haddad NS Anam FA Rudolph ME. Relaxed Peripheral Tolerance Drives Broad *De Novo* Autoreactivity in Severe Covid-19. Medrxiv (2021) 2020.10.21.20216192. doi: 10.1101/2020.10.21.20216192

[120] Nasserie T Hittle M Goodman SN. Assessment of the Frequency and Variety of Persistent Symptoms Among Patients With Covid-19: A Systematic Review. JAMA Network Open (2021) 4(5):e2111417–e. doi: 10.1001/jamanetworkopen.2021.11417 PMC8155823 34037731

[121] Mehandru S Merad M. Pathological Sequelae of Long-Haul Covid. Nat Immunol (2022) 23(2):194–202. doi: 10.1038/s41590-021-01104-y 35105985 PMC9127978

[122] Xie Y Al-Aly Z. Risks and Burdens of Incident Diabetes in Long Covid: A Cohort Study. Lancet Diabetes Endocrinol (2022) 10(5):311–21. doi: 10.1016/S2213-8587(22)00044-4 PMC8937253 35325624

[123] Gruber CN Patel RS Trachtman R Lepow L Amanat F Krammer F. Mapping Systemic Inflammation and Antibody Responses in Multisystem Inflammatory Syndrome in Children (Mis-C). Cell (2020) 183(4):982–95.e14. doi: 10.1016/j.cell.2020.09.034 32991843 PMC7489877

[124] Carter MJ Fish M Jennings A Doores KJ Wellman P Seow J. Peripheral Immunophenotypes in Children With Multisystem Inflammatory Syndrome Associated With Sars-Cov-2 Infection. Nat Med (2020) 26(11):1701–7. doi: 10.1038/s41591-020-1054-6 32812012

[125] Porritt RA Binek A Paschold L Rivas MN McArdle A Yonker LM. The Autoimmune Signature of Hyperinflammatory Multisystem Inflammatory Syndrome in Children. J Clin Invest (2021) 131(20):e151520. doi: 10.1172/jci151520 34437303 PMC8516454

[126] Consiglio CR Cotugno N Sardh F Pou C Amodio D Rodriguez L. The Immunology of Multisystem Inflammatory Syndrome in Children With Covid-19. Cell (2020) 183(4):968–81.e7. doi: 10.1016/j.cell.2020.09.016 32966765 PMC7474869

[127] Huang JJ Gaines SB Amezcua ML Lubell TR Dayan PS Dale M. Upregulation of Type 1 Conventional Dendritic Cells Implicates Antigen Cross-Presentation in Multisystem Inflammatory Syndrome. J Allergy Clin Immunol (2022) 149(3):912–22. doi: 10.1016/j.jaci.2021.10.015 PMC8530782 34688775

[128] Bernardes JP Mishra N Tran F Bahmer T Best L Blase JI. Longitudinal Multi-Omics Analyses Identify Responses of Megakaryocytes, Erythroid Cells, and Plasmablasts as Hallmarks of Severe Covid-19. Immunity (2020) 53(6):1296–314.e9. doi: 10.1016/j.immuni.2020.11.017 33296687 PMC7689306

[129] Rubtsova K Rubtsov AV Cancro MP Marrack P. Age-Associated B Cells: A T-Bet-Dependent Effector With Roles in Protective and Pathogenic Immunity. J Immunol (2015) 195(5):1933–7. doi: 10.4049/jimmunol.1501209 PMC4548292 26297793

[130] Rubtsov AV Rubtsova K Fischer A Meehan RT Gillis JZ Kappler JW. Toll-Like Receptor 7 (Tlr7)-Driven Accumulation of a Novel Cd11c_+_ B-Cell Population Is Important for the Development of Autoimmunity. Blood (2011) 118(5):1305–15. doi: 10.1182/blood-2011-01-331462 PMC3152497 21543762

[131] Rubtsova K Rubtsov AV Dyk LFV Kappler JW Marrack P. T-Box Transcription Factor T-Bet, a Key Player in a Unique Type of B-Cell Activation Essential for Effective Viral Clearance. Proc Natl Acad Sci (2013) 110(34):E3216–E24. doi: 10.1073/pnas.1312348110 PMC3752276 23922396

[132] Rubtsova K Rubtsov AV Thurman JM Mennona JM Kappler JW Marrack P. B Cells Expressing the Transcription Factor T-Bet Drive Lupus-Like Autoimmunity. J Clin Invest (2017) 127(4):1392–404. doi: 10.1172/JCI91250 PMC5373868 28240602

[133] Rivera-Correa J Rodriguez A. Divergent Roles of Antiself Antibodies During Infection. Trends Immunol (2018) 39(7):515–22. doi: 10.1016/j.it.2018.04.003 PMC6386177 29724608

[134] Pfeifer J Thurner B Kessel C Fadle N Kheiroddin P Regitz E. Autoantibodies Against Interleukin-1 Receptor Antagonist in Multisystem Inflammatory Syndrome in Children: A Multicentre, Retrospective, Cohort Study. Lancet Rheumatol (2022) 4(5):e329–37. doi: 10.1016/S2665-9913(22)00064-9 PMC8963770 35368387

[135] Elkon K Casali P. Nature and Functions of Autoantibodies. Nat Clin Pract Rheumatol (2008) 4(9):491–8. doi: 10.1038/ncprheum0895 PMC2703183 18756274

[136] Yonker LM Swank Z Gilboa T Senussi Y Kenyon V Papadakis L. Zonulin Antagonist, Larazotide (At1001), as an Adjuvant Treatment for Multisystem Inflammatory Syndrome in Children: A Case Series. Crit Care Explor (2022) 10(2):e0641. doi: 10.1097/cce.0000000000000641 35211683 PMC8860335

[137] LaRovere KL Riggs BJ Poussaint TY Young CC Newhams MM Maamari M. Neurologic Involvement in Children and Adolescents Hospitalized in the United States for Covid-19 or Multisystem Inflammatory Syndrome. JAMA Neurol (2021) 78(5):536–47. doi: 10.1001/jamaneurol.2021.0504 PMC7936352 33666649

[138] Chou SH-Y Beghi E Helbok R Moro E Sampson J Altamirano V. Global Incidence of Neurological Manifestations Among Patients Hospitalized With Covid-19—A Report for the Gcs-Neurocovid Consortium and the Energy Consortium. JAMA Network Open (2021) 4(5):e2112131–e. doi: 10.1001/jamanetworkopen.2021.12131 PMC8114143 33974053

[139] Khan M Yoo SJ Clijsters M Backaert W Vanstapel A Speleman K. Visualizing in Deceased Covid-19 Patients How Sars-Cov-2 Attacks the Respiratory and Olfactory Mucosae But Spares the Olfactory Bulb. Cell (2021) 184(24):5932–49.e15. doi: 10.1016/j.cell.2021.10.027 34798069 PMC8564600

[140] Douaud G Lee S Alfaro-Almagro F Arthofer C Wang C McCarthy P. Sars-Cov-2 Is Associated With Changes in Brain Structure in Uk Biobank. Nature (2022) 604(7907):697–707. doi: 10.1038/s41586-022-04569-5 35255491 PMC9046077

[141] Gollub RL. Brain Changes After Covid Revealed by Imaging. Nature (2022) 604(7907):633–4. doi: 10.1038/d41586-022-00503-x 35260835

[142] Fernández-Castañeda A Lu P Geraghty AC Song E Lee MH Wood J. Mild Respiratory Sars-Cov-2 Infection Can Cause Multi-Lineage Cellular Dysregulation and Myelin Loss in the Brain. bioRxiv (2022) 2022.01.07.475453. doi: 10.1101/2022.01.07.475453

[143] Villeda SA Luo J Mosher KI Zou B Britschgi M Bieri G. The Ageing Systemic Milieu Negatively Regulates Neurogenesis and Cognitive Function. Nature (2011) 477(7362):90–4. doi: 10.1038/nature10357 PMC3170097 21886162

[144] Iadecola C Anrather J Kamel H. Effects of Covid-19 on the Nervous System. Cell (2020) 183(1):16–27.e1. doi: 10.1016/j.cell.2020.08.028 32882182 PMC7437501

[145] Song E Bartley CM Chow RD Ngo TT Jiang R Zamecnik CR. Divergent and Self-Reactive Immune Responses in the Cns of Covid-19 Patients With Neurological Symptoms. Cell Rep Med (2021) 2(5):100288. doi: 10.1016/j.xcrm.2021.100288 33969321 PMC8091032

[146] Marshall M. Covid and the Brain: Researchers Zero in on How Damage Occurs (2021). Available at: https://www.nature.com/articles/d41586-021-01693-6. 10.1038/d41586-021-01693-6 34234323

[147] Franke C Ferse C Kreye J Reincke SM Sanchez-Sendin E Rocco A. High Frequency of Cerebrospinal Fluid Autoantibodies in Covid-19 Patients With Neurological Symptoms. Brain Behav Immun (2021) 93:415–9. doi: 10.1016/j.bbi.2020.12.022 PMC7834471 33359380

[148] Kreye J Reincke SM Prüss H. Do Cross-Reactive Antibodies Cause Neuropathology in Covid-19? Nat Rev Immunol (2020) 20(11):645–6. doi: 10.1038/s41577-020-00458-y PMC7537977 33024283

[149] Batisson M Strazielle N Hejmadi M Thomas D Ghersi-Egea JF Etienne J. Toxic Shock Syndrome Toxin-1 Challenges the Neuroprotective Functions of the Choroidal Epithelium and Induces Neurotoxicity. J Infect Dis (2006) 194(3):341–9. doi: 10.1086/505428 16826482

[150] Changeux JP Amoura Z Rey FA Miyara M. A Nicotinic Hypothesis for Covid-19 With Preventive and Therapeutic Implications. C R Biol (2020) 343(1):33–9. doi: 10.5802/crbiol.8 32720486

[151] Li Y Luo C Li W Xu Z Zeng C Bi S. Structure-Based Preliminary Analysis of Immunity and Virulence of Sars Coronavirus. Viral Immunol (2004) 17(4):528–34. doi: 10.1089/vim.2004.17.528 15671749

[152] Mateus J Grifoni A Tarke A Sidney J Ramirez SI Dan JM. Selective and Cross-Reactive Sars-Cov-2 T Cell Epitopes in Unexposed Humans. Science (2020) 370(6512):89–94. doi: 10.1126/science.abd3871 32753554 PMC7574914

[153] Buzhdygan TP DeOre BJ Baldwin-Leclair A Bullock TA McGary HM Khan JA. The Sars-Cov-2 Spike Protein Alters Barrier Function in 2d Static and 3d Microfluidic *in-Vitro* Models of the Human Blood-Brain Barrier. Neurobiol Dis (2020) 146:105131. doi: 10.1016/j.nbd.2020.105131 33053430 PMC7547916

[154] Rhea EM Logsdon AF Hansen KM Williams LM Reed MJ Baumann KK. The S1 Protein of Sars-Cov-2 Crosses the Blood-Brain Barrier in Mice. Nat Neurosci (2021) 24(3):368–78. doi: 10.1038/s41593-020-00771-8 PMC8793077 33328624

[155] Bollavaram K Leeman TH Lee MW Kulkarni A Upshaw SG Yang J. Multiple Sites on Sars-Cov-2 Spike Protein Are Susceptible to Proteolysis by Cathepsins B, K, L, S and V. Protein Sci (2021) 30(6):1131–43. doi: 10.1002/pro.4073 PMC8138523 33786919

[156] Swank Z Senussi Y Alter G Walt DR. Persistent Circulating Sars-Cov-2 Spike Is Associated with Post-Acute Covid-19 Sequelae. Medrxiv (2022) 2022.06.14.22276401. doi: 10.1101/2022.06.14.22276401

